# The Functional Role of Olfactory Bulb Granule Cell Subtypes Derived From Embryonic and Postnatal Neurogenesis

**DOI:** 10.3389/fnmol.2018.00229

**Published:** 2018-07-05

**Authors:** Hiroo Takahashi, Seiichi Yoshihara, Akio Tsuboi

**Affiliations:** ^1^Laboratory for the Molecular Biology of Neural Systems, Advanced Medical Research Center, Nara Medical University, Kashihara, Japan; ^2^Laboratory for the Molecular and Cellular Neuroscience, Graduate School of Frontier Biosciences, Osaka University, Suita, Japan

**Keywords:** neurogenesis, olfactory bulb interneuron, embryonic-born neurons, postnatal-born neurons, activity-dependent development

## Abstract

It has been shown in a variety of mammalian species that sensory experience can regulate the development of various structures, including the retina, cortex, hippocampus, and olfactory bulb (OB). In the mammalian OB, the development of dendrites in excitatory projection neurons, such as mitral and tufted cells, is well known to be dependent on odor experience. Odor experience is also involved in the development of another OB population, a subset of inhibitory interneurons that are generated in the ventricular-subventricular zone throughout life and differentiate into granule cells (GCs) and periglomerular cells. However, the roles that each type of interneuron plays in the control of olfactory behaviors are incompletely understood. We recently found that among the various types of OB interneurons, a subtype of GCs expressing the *oncofetal trophoblast glycoprotein 5T4* gene is required for odor detection and discrimination behaviors. Our results suggest that embryonic-born OB interneurons, including 5T4-positive GCs, play a crucial role in fundamental olfactory responses such as simple odor detection and discrimination behaviors. By contrast, postnatal- and adult-born OB interneurons are important in the learning of more complicated olfactory behaviors. Here, we highlight the subtypes of OB GCs, and discuss their roles in olfactory processing and behavior, with a particular focus on the relative contributions of embryonically and postnatally generated subsets of GCs in rodents.

## Introduction

Sensory experience plays a pivotal role in the development and plastic modification of neural circuitry in vertebrates (Katz and Shatz, 1996; Sanes and Lichtman, 2001; Nithianantharajah and Hannan, 2006; Lepousez et al., 2013). Specific odorants are detected by olfactory sensory neurons (OSNs) expressing corresponding odorant receptors in the olfactory epithelium (Mori and Sakano, 2011). Since OSN axons converge on specific glomeruli in the olfactory bulb (OB), OSNs can activate a specific neural circuit, and can also expedite dendritic development in a particular subset of interneurons via excitatory projection neurons within the OB (Mori and Sakano, 2011; Lepousez et al., 2013; Figueres-Oñate et al., 2014). The neural progenitor cells (NPCs) for OB interneurons are produced in the ventricular-subventricular zone (V-SVZ) of the lateral ventricle, in adulthood as well as in embryonic and neonatal stages (Chazal et al., 2000; Alvarez-Buylla et al., 2008; Lledo et al., 2008). OB interneuron NPCs move through the rostral migratory stream (RMS) to the OB, where they differentiate into γ-aminobutyric acid (GABA)-releasing inhibitory interneurons, including granule cells (GCs) and periglomerular cells (PGCs) (Lois and Alvarez-Buylla, 1994; Whitman and Greer, 2009; Adam and Mizrahi, 2010; Kaneko et al., 2010; Sequerra, 2014). In the OB, GCs, and PGCs form dendrodendritic reciprocal synapses with excitatory projection neurons including mitral and tufted cells (MCs/TCs); they receive glutamatergic inputs from the dendrites of MCs/TCs, and return the GABAergic outputs to the dendrites of MCs/TCs (Isaacson and Strowbridge, 1998; Shepherd et al., 2004; Whitman and Greer, 2007). Odor-evoked activity affects the survival of newborn OB interneurons and their integration into pre-existing neural circuits (Rochefort et al., 2002; Yamaguchi and Mori, 2005; Lin et al., 2010). Moreover, olfactory sensory deprivation and odor-rich environments can suppress or accelerate, respectively, the dendritic development and spine formation of newborn interneurons in the OB (Saghatelyan et al., 2005; Livneh et al., 2009). Despite continuous progress in the field, the roles that each type of newborn interneuron plays in the control of different behaviors remain poorly understood. In this review, we focus on the functional role of OB GC subtypes in olfactory processing and behavior.

## Embryonic and Postnatal Neurogenesis

In the rodent brain, OB interneurons are generated in the V-SVZ during both embryonic and postnatal stages. Embryonic neurogenesis starts with neuroepithelial cells located in the V-SVZ, and their transformation into radial glial cells (RGCs) (Alvarez-Buylla et al., 2001; Temple, 2001; Götz and Huttner, 2005; Kriegstein and Alvarez-Buylla, 2009). During this transformation, the neuroepithelial cells begin to lose certain epithelial properties such as tight junctions (Aaku-Saraste et al., 1997; Zhadanov et al., 1999), and instead acquire astroglial features, including the expression of several astrocyte markers (Hartfuss et al., 2001; Noctor et al., 2002). Many intrinsic signals act synergistically to support this transition and ensure robust embryonic neurogenesis (Götz and Huttner, 2005; Kriegstein and Alvarez-Buylla, 2009). RGCs initially work as fate-restricted NPCs that either directly produce nascent neurons or generate neuronal intermediate progenitor cells (IPCs), which in turn differentiate into neurons through symmetrical mitosis (Götz and Huttner, 2005; Turrero-García and Harwell, 2017). In late development, RGCs also produce glial cells such as astrocytes and oligodendrocytes (Mission et al., 1991; Merkle et al., 2004; Noctor et al., 2008).

**FIGURE 1:**
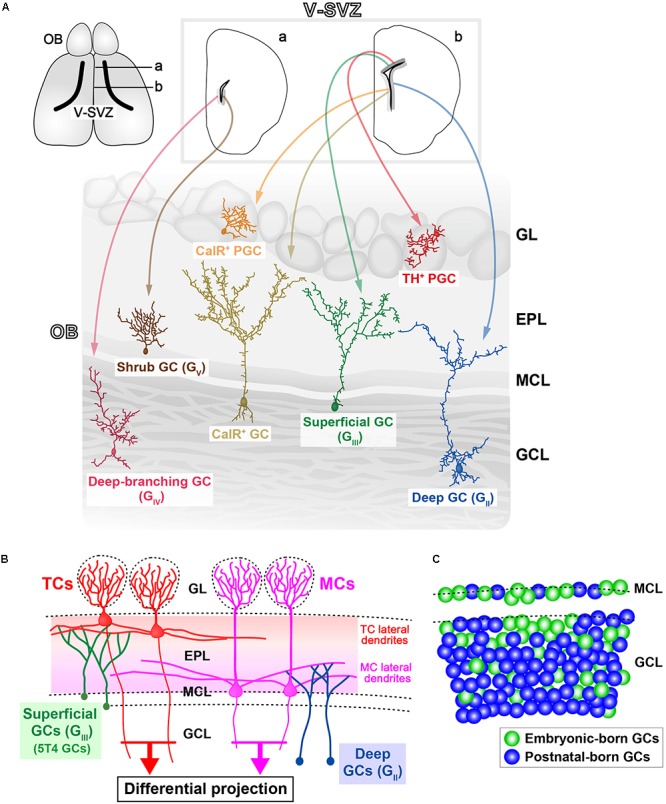
Various subtypes of olfactory bulb (OB) interneurons. **(A)** Regional organization of neural stem cells (NSCs) in the ventricular-subventricular zone (V-SVZ). OB interneurons are generated in distinct subregions of the adult V-SVZ (*top sections*; **a**,**b**), and then differentiate into unique types of interneurons in the OB, such as granule cells (GCs) and periglomerular cells (PGCs) (*bottom section*). As examples, two subtypes of PGCs [calretinin (CalR), tyrosine hydroxylase (TH)], three subtypes of GCs (superficial, deep, and CalR) (Merkle et al., 2007), and two novel subtypes of GCs (deep-branching and shrub) (Merkle et al., 2014) are shown. Note that dopaminergic neurons in the glomerular layer (indicated as TH^+^ PGC) are also referred to as short-axon (SA) cells (Kiyokage et al., 2010). GL, glomerular layer; EPL, external plexiform layer; MCL, mitral cell layer; GCL, granule cell layer. **(B)** Schematic representation of the OB neural circuitry. Superficial GCs (G_III_), including 5T4 GCs, preferentially target the lateral dendrites of tufted cells (TCs) within the upper EPL. However, deep GCs (G_II_) mainly connect to MCs within the deep EPL. Parallel TC and mitral cell (MC) pathways route distinct odor information to different areas of the olfactory cortex. Therefore, it is likely that each GC subtype plays a distinct role in odor processing. **(C)** Schematic representation of integration modes for embryonic-born (*green*) and postnatal-born (*blue*) GCs in the OB. In adult mice, the dynamic turnover of old GCs and newborn GCs occurs continuously. It is suggested that postnatal-born GCs are integrated preferentially into the deeper GC layers in the OB (Sakamoto et al., 2014).

In the adult V-SVZ, radial glia-like neural stem cells (NSCs; also called type B cells) produce different types of adult-born interneurons that migrate to the OB (Lois and Alvarez-Buylla, 1994; Doetsch et al., 1999). Adult NSCs are generated from RGCs (Merkle et al., 2004; Bonfanti and Peretto, 2007), and different types of OB interneurons are derived from the NSCs, according to their position in the V-SVZ (Merkle et al., 2007, 2014; **Figure 1A**). However, it is unknown when this spatial determination of cell fate occurs, and whether adult NSCs are derived from the same neural precursors that account for embryonic neurogenesis. It is reported that adult NSCs have an embryonic origin; adult NSC precursors are generated between embryonic day (ED)13.5 and ED15.5, but remain relatively quiescent until being reactivated postnatally (Fuentealba et al., 2015; Furutachi et al., 2015). In other words, the majority of RGCs terminally differentiate into neuronal and glial cells by the end of development [postnatal day (PD)15; Tramontin et al., 2003], while a small population of NSCs remain quiescent through embryonic development. These residual NSCs are responsible for adult V-SVZ neurogenesis: they can be activated to produce IPCs, which in turn generate neuroblasts. Neuroblasts and their immature neuronal progeny migrate in chains through the RMS to the OB, where they then differentiate into OB interneurons.

In adult rodents, the RMS carries considerable numbers of neuroblasts into the OB (Lois and Alvarez-Buylla, 1994; Alvarez-Buylla et al., 2001). Bromodeoxyuridine (BrdU) labeling in adult mice (8 weeks old) indicated that more than 20,000 newborn cells had reached the OB 14 days after a BrdU injection (Yamaguchi and Mori, 2005), thereby demonstrating the robust neurogenesis and large-scale plasticity occurring in this region. Furthermore, local cell proliferation in the OB has also been reported, especially within the early postnatal days (PD3) (Lemasson et al., 2005).

## Subtypes of OB GCs

Granule cells have a small soma without any axons, and form dendrodendritic synapses with excitatory projection neurons (Price and Powell, 1970a,b; Shepherd et al., 2007). GCs comprise the largest population of neurons in the OB. Most (>95%) of the adult-born neurons in the V-SVZ differentiate into GCs in the OB (Winner et al., 2002). First, GCs are classified into several subtypes according to their dendritic morphologies and the locations of their cell bodies within the GC layer. Horseradish peroxidase injection and Golgi staining indicate that GCs in rodents can be divided into three subtypes: intermediate (G_I_), deep (G_II_), and superficial (G_III_) GCs (Mori et al., 1983; Orona et al., 1983; Greer, 1987; Shepherd et al., 2004). Recent studies also show that there are further subtypes of GCs, such as deep-branching GCs (G_IV_), shrub GCs (G_V_) (Merkle et al., 2014), and type-S GCs (Naritsuka et al., 2009). Second, GCs are also classified according to their gene expression: *oncofetal trophoblast glycoprotein 5T4* and *Calretinin* (*CalR*) are expressed in unique subtypes of OB GCs, termed 5T4 GCs and CalR GCs, respectively (Jacobowitz and Winsky, 1991; Imamura et al., 2006; Parrish-Aungst et al., 2007). Both of these subtypes are located in the MC and superficial GC layers (Imamura et al., 2006; Parrish-Aungst et al., 2007). 5T4 GCs seem to be the subpopulation in superficial GCs (G_III_), based on their locations and their dendritic morphologies (Imamura et al., 2006). In contrast, NSCs producing CalR GCs are different from those generating superficial GCs (G_III_) (Merkle et al., 2007; **Figure 1A**). GCs can be divided into metabotropic glutamate receptor (mGluR2)-positive (one-third of the total GCs) and -negative subsets (two-thirds) throughout the GC layer (Ohishi et al., 1993, 1998; Murata et al., 2011). The Ca^2+^/calmodulin-dependent protein kinases *Camk2a* (Zou et al., 2002; Malvaut et al., 2017) and *Camk4* (Baker et al., 2001), and the transcription factor *ER81* (Stenman et al., 2003), are also expressed in subpopulations of GCs.

**FIGURE 2:**
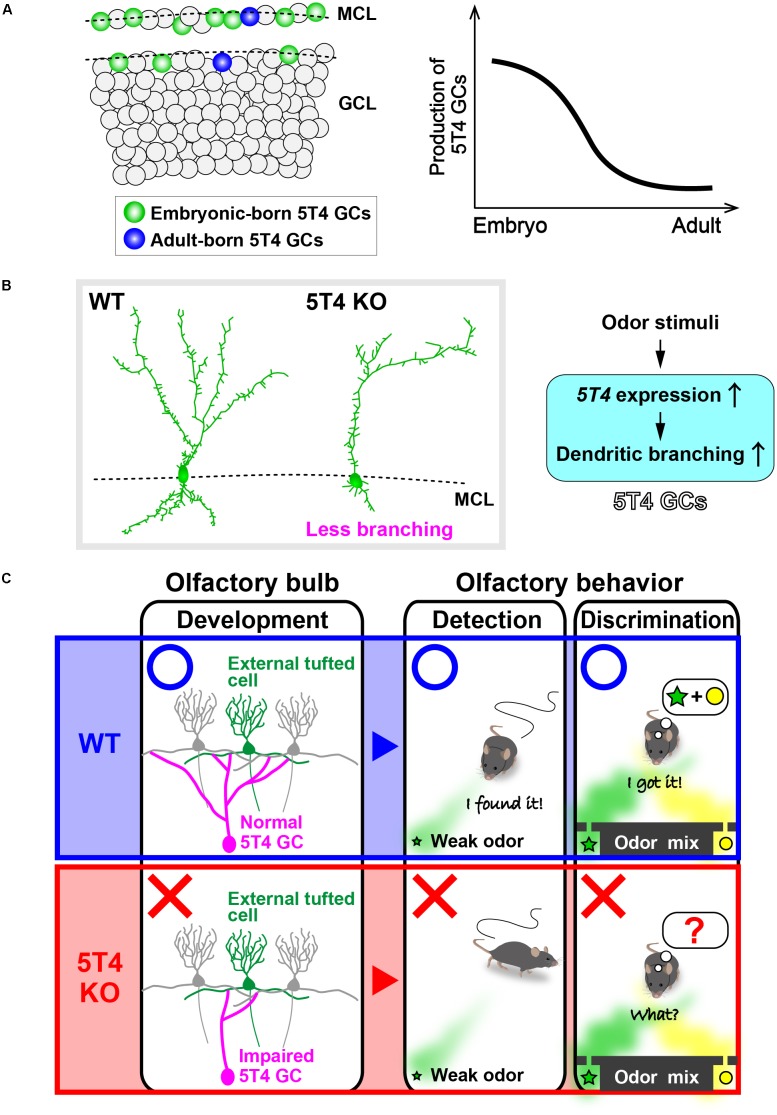
5T4 granule cells (GCs) are required for odor detection and discrimination behaviors. **(A)** Production of 5T4 GCs in embryonic and adult stages. Bromodeoxyuridine (BrdU) labeling indicates that 5T4 GCs are generated mainly during the embryonic stage (*green*), rather than in the adult stage (*blue*) (Takahashi et al., 2016). MCL, mitral cell layer; GCL, granule cell layer. **(B)** Dendritic branching in 5T4 GCs. *5T4* knock-out (KO) mice showed significantly reduced dendritic branching from 5T4 GCs (Yoshihara et al., 2012), with *5T4* being necessary for the dendritic branching in 5T4 GCs. **(C)** Schematic representation of the impairment in *5T4* KO mice. The dendrodendritic synapses between 5T4 GCs and the external TCs were significantly decreased in *5T4* KO mice. *5T4* KO mice cannot detect a low concentration of odorants or discriminate pairs of odorants, while wild-type (WT) mice are able to. These findings therefore imply that 5T4 GCs are required for odor detection and discrimination behaviors.

As described above, adult NSCs in different subregions of the V-SVZ appear to produce distinct types of OB interneurons, including a variety of GC subtypes (Merkle et al., 2007, 2014; **Figure 1A**). Until recently, it remained unknown whether this spatial determination of cell fate is established in early development. A genetic barcode technique for tracing the lineage of cells has revealed that different types of adult-born OB interneurons correlate with distinct classes of forebrain neurons, according to their position in the V-SVZ (Fuentealba et al., 2015). For example, superficial GCs and dopaminergic PGCs, which are produced in the dorsal V-SVZ in adulthood, are clonally related to cortical neurons generated in the corresponding region during the embryonic stage. This result suggests that in the common progenitors for the forebrain NSCs and adult NSCs, the spatial determination of cell fate is established at the early embryonic stage. In addition, distinct subsets of OB GCs are preferentially produced during different stages. Notably, although 5T4 GCs are generated from the embryonic (ED12.5) to postnatal stages (PD30) (Batista-Brito et al., 2008), these production is mainly during the embryonic (ED15.5) and neonatal (PD0) stages, rather than during the adult stage (PD56) (Sakamoto et al., 2014; Takahashi et al., 2016; **Figure 2A**). Similarly, CalR GCs, which are located in the superficial GC layer, are remarkably produced just after birth (PD0) (Batista-Brito et al., 2008). The tracing of the genetic lineage of postnatal-born neurons is performed using *ROSA26* reporter mice crossed with *GFAP* promoter–*Cre* mice (Sakamoto et al., 2014), in which *Cre* recombinase is expressed in the postnatal NSCs (Garcia et al., 2004). Interestingly, nearly 90% of the GCs are labeled as postnatal-born neurons in the deep part of the GC layer in the adult mice (PD90), whereas only about 40 and 60% of GCs are labeled in the MC and superficial GC layers, respectively. These results suggest that embryonically derived GCs tend to be maintained in the MC and superficial GC layers, whereas postnatal-born neurons are integrated predominantly into the deep GC layer (Sakamoto et al., 2014; **Figure 1C**). To confirm this, Imayoshi et al. (2008) performed the genetic ablation of adult-born neurons using tamoxifen-inducible *diphtheria toxin fragment A* (*DTA*) mice. In their study, *Nestin* promoter–tamoxifen-inducible *Cre* (*CreER^T2^*) mice (Imayoshi et al., 2006) were crossed with *neuron-specific enolase 2 (NSE)* promoter–*floxed*-*STOP*-*DTA* mice (Kobayakawa et al., 2007). Tamoxifen treatment of the adult transgenic mice (2-months-old) induced recombination in the adult NSCs, and *DTA* was expressed in the neurons differentiated from the adult NSCs. The ablation of many newborn neurons in the OB started around 3 weeks after the tamoxifen treatment (Imayoshi et al., 2008). Interestingly, the genetic ablation of newborn neurons in adult mice results in a lack of GCs, particularly in the deep GC layer, indicating that continuous adult neurogenesis is required for the maintenance and reorganization of GCs, especially in the deep GC layer (Imayoshi et al., 2008). These results indicate that the embryonic, neonatal, and/or adult stages show different tendencies in the GC subtypes generated (Lemasson et al., 2005).

When a GC subtype is impaired, it is possible that the same subtype of newborn GCs are replaced in the OB circuit. For example, selective ablation of *mGluR2*-expressing GCs tends to result in recruitment of new mGluR2-positive GCs, as opposed to mGluR2-negative GCs (Murata et al., 2011). A similar result is also reported for the other type of OB interneurons, PGCs: when tyrosine hydroxylase (TH)-positive PGCs are laser-ablated at a specific position in the glomerular layer, newly generated TH-positive PGCs are integrated into the same position (Sawada et al., 2011). These results suggest that the impaired area of the OB can cue the replacement of a specific subset of interneurons by incorporating newborn interneurons into the circuit. Notably, the adult neurogenesis in the V-SVZ OB is clearly different from that in the hippocampus, where newly generated neurons are not necessary for maintenance of the total number of dentate gyrus neurons in adulthood (Imayoshi et al., 2008).

Questions can then be asked as to what distinct roles newborn OB GCs play in the processing of odor information and how the roles depend on the subset variety. OB GCs are inhibitory interneurons and control the neural activity of excitatory projection neurons, regulating the odor information being sent to the higher brain center, olfactory cortex (OC). The role of each subset of GCs in odor processing depends on the type of excitatory projection neurons the subset connects to, as well as the synaptic loci and numbers. Additionally, the functioning of each GC subtype is also affected by other inhibitory interneurons and centrifugal inputs from outside the OB (Pressler and Strowbridge, 2006; Nunez-Parra et al., 2013).

## Neural Circuits Between GCs and Projection Neurons

The synchronized firing of MCs/TCs gives rise to prominent γ-range oscillations in the local field potential, with these being necessary for effective transmission of odor information to the OC in awake behaving animals (Beshel et al., 2007; Manabe and Mori, 2013). The γ-range oscillations in the OB are generated by dendrodendritic synapses between MCs/TCs and GCs (Nusser et al., 2001; Lagier et al., 2004). MCs preferentially elongate their lateral dendrites within the deeper external proximal layer (EPL), while TCs elongate within the upper EPL. It was reported that local dendrodendritic circuits within the EPL may segregate into multiple sublayers (Orona et al., 1984; Mori et al., 2013; Fourcaud-Trocmé et al., 2014). Superficial GCs (G_III_) mainly branch their dendrites in the superficial EPL, among the lateral dendrites of TCs (Mori et al., 1983; Orona et al., 1983; Greer, 1987; Shepherd et al., 2004; **Figure 1B**). In particular, 5T4 GCs have dendrites that branch highly within the superficial EPL (Imamura et al., 2006; Yoshihara et al., 2012). Deep GCs (G_II_) mainly extend their dendrites to the deep EPL, where they are among the dendrites of MCs (**Figure 1B**), while intermediate GCs (G_I_) have dendrites that ramify at all levels of the EPL. It thus appears that MCs and TCs have both segregated and overlapping microcircuits via GCs (Macrides et al., 1985).

Igarashi et al. (2012) reported that TCs show odor-induced short-latency firing responses for a wide range of odor concentrations, whereas MCs respond only to strong signals. Optogenetic activation of a single glomerulus has revealed that lateral inhibition only affects TCs when they are firing at low rates, whereas MCs are affected by lateral inhibition when firing at intermediate rates (Geramita et al., 2016). Geramita et al. (2016) proposed that differences in the lateral inhibition between MCs and TCs are likely to result from their different connections to distinct GC subtypes such as the deep and superficial GCs. Furthermore, MCs receive stronger inhibitory currents from glomerular layer circuits than TCs (Geramita and Urban, 2017). In addition, individual TCs demonstrate dense projections to anterior areas of the OC, whereas individual MCs show dispersed projections to all OC areas (Nagayama et al., 2010; Miyamichi et al., 2011; Fukunaga et al., 2012; Igarashi et al., 2012). These studies suggest that MCs and TCs transmit temporally different odor signals to distinct areas of the OC. It is possible that each GC subtype plays a distinct role in OB circuitry through the regulation of MCs, TCs, or both (Shepherd et al., 2004).

## Roles of OB Interneurons in Olfactory Behaviors

To reveal the function of OB interneurons in olfactory behaviors, previous studies used mutant mice with impaired OB interneurons (Gheusi et al., 2000; Kim et al., 2007; Bath et al., 2008). Impairment of odor discrimination is observed in both *neural cell adhesion molecule* (*NCAM*)-deficient mice (Gheusi et al., 2000) and *brain derived neurotrophic factor* (*Bdnf*)-mutant mice (Bath et al., 2008). Pharmacological approaches have been used to directly control adult neurogenesis, including the repression of adult neurogenesis by the antimitotic drug cytosine arabinoside (Ara-C), which results in impairments to both the sensitivity of odor detection and the retention of olfactory memory (Breton-Provencher et al., 2009; Sultan et al., 2010). Additionally, the use of the caspase inhibitor benzyloxycarbonyl-Val-Ala-Asp (ZVAD) to repress cell death in OB interneurons is reported to result in increased reaction times for olfactory discrimination (Mouret et al., 2009), and yet also to facilitate the retention of olfactory memory (Sultan et al., 2011). It is possible that differences in the ZVAD administration protocol (e.g., duration and timing) can give rise to opposing effects (decline and improvement) in behavioral tests. In the least, these pharmacological studies suggest that the generation of OB newborn neurons and their appropriate elimination are required for various olfactory behaviors such as odor detection, odor discrimination, and the retention of olfactory memory. Although these pharmacological approaches are useful for studying the role of adult neurogenesis, it is difficult to specifically target newborn neurons, because neurons and glia in other cortical regions may also be affected by the drugs. Irradiation specific to the V-SVZ represses adult neurogenesis and impairs long-term olfactory memory (Lazarini et al., 2009). However, it also seems to be difficult to achieve specific and complete inhibition of adult neurogenesis using this approach.

To resolve this problem, genetic ablation of newborn neurons can be performed using tamoxifen-inducible *DTA* mice (Imayoshi et al., 2008; Sakamoto et al., 2011). Surprisingly, the ablation of newly born neurons in adult mice affects innate olfactory responses such as predator avoidance and sex-specific responses, but not simple olfactory associative learning of a pair of optically isomeric odorants. Tamoxifen-inducible *DTA* mice show a reduction of newborn neurons not only in the OB, but also in the hippocampus and accessory OB (Sakamoto et al., 2011), with the latter playing a crucial role in the processing of pheromone information. It is therefore uncertain whether the reduction of newborn OB interneurons affects innate olfactory responses. To increase the target specificity, Sakamoto et al. (2014) performed an intersectional strategy with dual site-specific recombinases (Cre and Flp) and inhibited neuronal activity in the postnatal-born interneurons in the OB, but not in the hippocampus. Both Cre- and Flp-inducible *tetanus toxin light chain (TeNT)* mice (Sakamoto et al., 2014) have been crossed with transgenic mice expressing both *Cre* in the postnatal NSCs (Garcia et al., 2004) and *Flp* in the forebrain GABAergic neurons (Miyoshi et al., 2010). When the odorant (+)-carvone was associated with a sugar reward, these transgenic mice could learn and discriminate (+)-carvone in the presence of its optical isomer (-)-carvone, a finding that is consistent with a previous study by Imayoshi et al. (2008). However, after switching the sugar-associated odorant from (+)-carvone to (-)-carvone, they showed defects in the reverse-learning compared with wild-type mice (Sakamoto et al., 2014). These results suggest that postnatal-born OB interneurons are not necessary for simple odor sensing, but are important for flexible olfactory associative learning and memory.

Optogenetics is also a powerful tool in the field of OB neurogenesis. Lentiviral vectors carrying *channelrhodopsin-2* (*ChR2*) gene under the control of *Synapsin 1 (Syn1)* promoter have been injected into the lateral ventricles in adult stage mice (Alonso et al., 2012). A miniature LED was then positioned on top of a cranial window overlaying the dorsal OB. *In vivo* light activation (40 Hz, 5 ms, 20 pulses) of adult-born neurons paired with odor stimulation accelerated the olfactory discrimination learning rate in a difficult task (using a pair of similar odors), but not in an easy task (Alonso et al., 2012). The authors proposed a mechanism in which light stimulation activates the adult-born neurons (especially GCs) and produces a net inhibition of low-responding MCs (but not highly excited MCs), thereby facilitating the olfactory discrimination learning. Similar improvements to the learning of odor discrimination have also been observed in experiments in which adeno-associated virus (AAV) vectors carrying *ChR2* under the control of *CAG* promoter were injected into the GC layer of the OB (Gschwend et al., 2015). In this case, light stimulation (40 Hz, 5 ms, 80 pulses) through a cranial window seemed to activate both embryonic-born and postnatal-born GCs. Conversely, inhibition of neuronal activity in the OB GCs using *Designer Receptors Exclusively Activated by Designer Drugs (DREADDs)* genes under the control of *Syn1* promoter reduced the learning rate of olfactory discrimination (Gschwend et al., 2015). Consistent with findings following manipulations of postnatal-born interneurons (Imayoshi et al., 2008; Sakamoto et al., 2014), the results from manipulations of adult-born GCs (Alonso et al., 2012; Gschwend et al., 2015) suggest that adult-born OB GCs are both necessary and sufficient to improve learned olfactory behaviors. Interestingly, when lentiviral vectors carrying *ChR2* were injected into neonatal mice (PD6), a light stimulus applied to the adult mice to stimulate the early postnatal-born GCs did not improve learning rate (Alonso et al., 2012). These results therefore indicate specific behavioral effects resulting from the recruitment of newly generated GCs in adulthood, but not that of newborn GCs in early postnatal development.

The role of GCs in olfactory behaviors has also been studied using conditional *GABA_A_R β3-subunit* (*Gabrb3*) knock-out (KO) mice created by the injection of AAV vectors into the GC layer (Nunes and Kuner, 2015). These conditional *Gabrb3* KO mice showed reduced GABA_A_R-mediated inhibitory postsynaptic currents in GCs and increased recurrent inhibition in MCs. The resulting enhancement of neural activity in part of the embryonic- and postnatal-born GCs shortened the time required to discriminate both dissimilar and highly similar odors, while the learning of discrimination remained unaffected (Nunes and Kuner, 2015). It would be interesting to know why the enhancement of GC activity (derived from adult-born GCs) in the conditional *Gabrb3* KO mice did not improve the learning of odor discrimination, a finding that is in contrast to the results of optogenetic approaches (Alonso et al., 2012; Gschwend et al., 2015).

Despite continuous progress, as described above, the roles played by each subtype of newborn OB GCs in the control of olfactory behaviors remain poorly understood. Furthermore, it is uncertain whether embryonic-born GCs have a specific function. To address these issues, it is necessary to manipulate a specific subtype of OB GCs.

## A Subtype of OB GCs Expressing 5T4

The *oncofetal trophoblast glycoprotein 5T4* gene is expressed in a specific subtype of GCs named 5T4 GCs, which are located in the MC and superficial GC layers (Imamura et al., 2006; Yoshihara et al., 2012). The single-pass transmembrane protein 5T4 was identified while searching for molecules with invasive properties shared by cancer cells and placental trophoblasts (Hole and Stern, 1990). Expression of *5T4* is increased in many cancer cells: it is high in the brain and ovaries (King et al., 1999; Barrow et al., 2005), but only found at low levels in most normal tissues (Southall et al., 1990). In odor-deprived OB, both the number of 5T4 GCs and the expression of *5T4* by each 5T4 GC are decreased, thereby indicating that the expression of *5T4* by 5T4 GCs requires sensory inputs (Imamura et al., 2006; Yoshihara et al., 2012). However, it is uncertain whether odor enrichment facilitates *5T4* expression in 5T4 GCs. *5T4* KO and OB-specific knock-down (KD) mice show significantly reduced dendritic branching in 5T4 GCs, but not in the other GC subtypes (Yoshihara et al., 2012; Takahashi et al., 2016; **Figure 2B**). Therefore, *5T4* mutant mice are a good model for studying the function of a specific subtype of GCs in relation to olfactory behaviors. Interestingly, both *5T4* KO and KD mice have higher odor detection thresholds than wild-type mice, as well as defects in odor discrimination (Takahashi et al., 2016; **Figure 2C**), which thereby indicate that 5T4 GCs are required for odor detection and discrimination behaviors. The dendrites of 5T4 GCs are highly branching, especially in the superficial part of the EPL (Imamura et al., 2006; Yoshihara et al., 2012), and the *5T4* KO mice show a corresponding reduction in dendrodendritic synapses between 5T4 GCs and external TCs (ETCs, a subtype of TCs) (Takahashi et al., 2016; **Figure 2C**). It is thus speculated that 5T4 GCs preferentially adjust the TC pathway to control both odor detection thresholds and simple odor discrimination. Therefore, to clarify the role of 5T4 GCs in olfactory behavior, it is necessary to perform further analysis of such behavior by controlling neuronal activity with optogenetic and chemogenetic methods.

## Embryonic- and Postnatal-Born OB GCs

Granule cell-specific *Gabrb3* KO and OB-specific *5T4* KD mice show effects on their fundamental olfactory responses, such as odor discrimination and detection (Nunes and Kuner, 2015; Takahashi et al., 2016). However, neither inhibition nor activation of neural activity in postnatal-born OB interneurons, including GCs, has any remarkable effect on either odor detection thresholds or simple odor discrimination (Abraham et al., 2010; Alonso et al., 2012; Sakamoto et al., 2014). These discrepancies could be attributed to a difference in the GC subtypes that were genetically manipulated in each study. Sakamoto et al. (2014) proposed that ∼25% of GCs in adult mice are generated from embryonic NSCs, while other authors suggest that postnatal neurogenesis reaches a peak at PD7, and then declines to one-third by PD60 (Lemasson et al., 2005). In adult mice (PD90), nearly 90% of GCs in the deep regions of the GC layer are generated after birth (Sakamoto et al., 2014). Therefore, it is likely that manipulated adult-born GCs are preferentially integrated into the deep GC layer, and that these tend to connect to MCs rather than to TCs (Bardy et al., 2010). By contrast, embryonic-born GCs tend to be maintained in the MC and superficial GC layers (about 60 and 40% of the GCs, respectively) in adult mice (PD90). Consistent with these findings, 5T4 GCs, which are produced mainly during the embryonic and neonatal stages, are located in the MC and superficial GC layers (Sakamoto et al., 2014; Takahashi et al., 2016). Likely, in the GC-specific *Gabrb3* KO mice created by the injection of AAV vectors into the OB, only a part of embryonic- and postnatal-born GCs is manipulated (Nunes and Kuner, 2015). It was recently reported that another subtype of OB GCs, Camk2a^+^ GCs, which are located throughout the GC layer, are required for both spontaneous odor discrimination and the learning of odor discrimination (Malvaut et al., 2017).

The olfactory system is essential for the survival of newborn rodents, providing vital environmental information. Anosmia in rodents usually causes death shortly after birth, apparently because of a failure to feed (Singh et al., 1976; Nassar et al., 2004). Therefore, the OB neural circuit needs to correctly process environmental information immediately after birth. We speculate that embryonic-born GCs, including 5T4 GCs, play a role in the fundamental olfactory responses (odor detection and simple odor discrimination) that are important for survival, such as suckling behavior. By contrast, adult-born GCs are more important for learned olfactory behaviors (Alonso et al., 2012; Sakamoto et al., 2014). Consistent with these findings, projection neuron activity induces the relocation of mature spines in adult-born GCs, but not in early postnatal-born GCs (Breton-Provencher et al., 2016). If embryonic- and postnatal-born GCs play different roles in olfactory processing, it is assumed that they may result from the distinct subpopulations of interneurons that are generated at each stage (Lemasson et al., 2005; Batista-Brito et al., 2008; Sakamoto et al., 2014). Therefore, we should further analyze this from the viewpoint of interneuron types and their connecting projection neurons.

## Perspectives

Recent studies reveal that a variety of OB interneurons are generated in a temporally relevant manner from NSCs located on distinct subregions of the V-SVZ (Merkle et al., 2014). GC subtypes generated at the embryonic, neonatal, and adult stages show specific tendencies (Batista-Brito et al., 2008; Sakamoto et al., 2014). If each OB GC subtype plays a different role (Takahashi et al., 2016; Malvaut et al., 2017), the differences in the subpopulations of interneurons generated between the embryonic- and neonatal stages inevitably infer differential roles for them in olfactory processing. The roles of specific types of OB interneuron subsets are assumed to depend on the kinds of excitatory projection neurons to which a given OB interneuron connects, and also factors such as the synaptic locus and number of them. It is suggested that MCs and TCs transmit temporally distinct odor signals to different OC targets (Nagayama et al., 2010; Miyamichi et al., 2011; Fukunaga et al., 2012; Igarashi et al., 2012). Therefore, to reveal the distinct role that each subtype of GCs plays in the OB circuitry, it is important to perform further studies on the functional diversity between the MCs and TCs in respect to odor detection, odor discrimination, and odor-associated learning. In addition, it is suggested that the plasticity of each newborn GC is affected by its functional maturity (immature or mature). Immature GCs show a high level of filopodia formation and retraction on the distal dendrites, while these dendritic dynamics decrease as the GCs mature (Breton-Provencher et al., 2014). Similar findings are also reported for the other type of OB interneurons, the PGCs: younger PGCs also show remarkable dendritic dynamics in comparison with older ones (Livneh et al., 2009, 2014). We recently elucidated the mechanism regulating spine formation, specifically in younger GCs (Yoshihara et al., 2016). The transcription factor *Neuronal Per-Arnt-Sim domain protein 4* (*Npas4*) is expressed in the OB GCs in an activity-dependent manner (Bepari et al., 2012). Npas4 prevents degradation of the microtubule-associated protein doublecortin (Dcx), which facilitates migration and spine formation in newborn GCs (Belvindrah et al., 2011; Yoshihara et al., 2014). Since GCs decrease *Dcx* expression according to their maturation (Brown et al., 2003), Npas4 facilitates spine formation in immature GCs in an activity-dependent manner, but does not do this in mature GCs (Yoshihara et al., 2014). These results suggest that the maturity of each GC is also an important factor in the study of their function. To understand the role of each type of OB interneuron in olfactory processing and behavior, we should further analyze them from the viewpoints of (1) the variety of different types, (2) their birth time (e.g., embryonic- and postnatal-born), (3) their functional maturity (immature and mature), and (4) their connecting projection neurons (e.g., MT, TC, and ETC). For this purpose, it is necessary to identify a marker gene and its promoter that are specific for the distinct subset of OB interneurons, to allow manipulation of those neurons alone. Future studies on the roles of embryonic- and postnatal-born interneurons will shed light on the biological significance and necessity of adult neurogenesis in the V-SVZ OB system.

## Author Contributions

HT and SY constructed the figures. HT and AT wrote the paper.

## Conflict of Interest Statement

The authors declare that the research was conducted in the absence of any commercial or financial relationships that could be construed as a potential conflict of interest.
